# Follistatin, an Activin Antagonist, Ameliorates Renal Interstitial Fibrosis in a Rat Model of Unilateral Ureteral Obstruction

**DOI:** 10.1155/2014/376191

**Published:** 2014-05-05

**Authors:** Akito Maeshima, Keiichiro Mishima, Shin Yamashita, Masao Nakasatomi, Masaaki Miya, Noriyuki Sakurai, Toru Sakairi, Hidekazu Ikeuchi, Keiju Hiromura, Yoshihisa Hasegawa, Itaru Kojima, Yoshihisa Nojima

**Affiliations:** ^1^Department of Medicine and Clinical Science, Gunma University Graduate School of Medicine, Maebashi 371-8511, Japan; ^2^School of Veterinary Medicine and Animal Science, Kitasato University, Towada 034-8628, Japan; ^3^Institute for Molecular and Cellular Regulation, Gunma University, Maebashi 371-8512, Japan

## Abstract

Activin, a member of the TGF-*β* superfamily, regulates cell growth and differentiation in various cell types. Activin A acts as a negative regulator of renal development as well as tubular regeneration after renal injury. However, it remains unknown whether activin A is involved in renal fibrosis. To clarify this issue, we utilized a rat model of unilateral ureteral obstruction (UUO). The expression of activin A was significantly increased in the UUO kidneys compared to that in contralateral kidneys. Activin A was detected in glomerular mesangial cells and interstitial fibroblasts in normal kidneys. In UUO kidneys, activin A was abundantly expressed by interstitial *α*-SMA-positive myofibroblasts. Administration of recombinant follistatin, an activin antagonist, reduced the fibrotic area in the UUO kidneys. The number of proliferating cells in the interstitium, but not in the tubules, was significantly lower in the follistatin-treated kidneys. Expression of *α*-SMA, deposition of type I collagen and fibronectin, and CD68-positive macrophage infiltration were significantly suppressed in the follistatin-treated kidneys. These data suggest that activin A produced by interstitial fibroblasts acts as a potent profibrotic factor during renal fibrosis. Blockade of activin A action may be a novel approach for the prevention of renal fibrosis progression.

## 1. Introduction

Renal interstitial fibrosis is a common feature in various kidney diseases and correlates with renal dysfunction. The histological characteristics of renal fibrosis are excessive deposition of extracellular matrix (ECM) and accumulation of interstitial fibroblasts that proliferate, differentiate into myofibroblasts, and actively synthesize ECM [1]. During renal fibrosis, tubular epithelial cells were considered to transdifferentiate into interstitial fibroblasts via epithelial to mesenchymal transition [2, 3]. Transforming growth factor-beta 1 (TGF-*β*1), which shows enhanced expression in human fibrotic kidneys and in animal models of renal fibrosis, promotes renal fibrosis through the activation of interstitial fibroblasts and acts as a potent inducer of EMT [3, 4]. Blockade of TGF-*β*1 signals has been shown to ameliorate renal interstitial fibrosis in several experimental models [5]. However, the factors that contribute to renal fibrosis have not been fully identified.

Activin A, a member of the TGF-*β* superfamily, is a dimeric protein composed of two *β*A subunits and modulates cell growth and differentiation in various tissues. Activin exerts its biological effects by interacting with two types of transmembrane receptors (type I and type II) with intrinsic serine/threonine kinase activity [6]. A key regulatory factor that modulates activin A action is follistatin. Follistatin binds to activin A with high affinity and blocks its action [7]. Follistatin is synthesized in the target cells of activin A and remains in the extracellular matrix [8], while activin A is trapped by follistatin, internalized by endocytosis, and subsequently degraded by proteolysis [9].

Activin A acts as a negative regulator of renal organogenesis [10]. In the embryonic kidney, activin A suppresses branching of the ureteric bud and induces cell differentiation in the metanephric mesenchyme. Activin A is an endogenous inhibitor of ureteric bud formation from the Wolffian duct. Cancellation of the autocrine action of activin A may be critical for the initiation of this process. Transgenic mice expressing mutant activin receptor had an increased number of glomeruli in the kidney. Activin A inhibited three-dimensional tubular formation in an in vitro tubulogenesis model using MDCK cells. Activin A is also involved in the recovery process of the kidney after injury [11]. Expression of activin A was undetectable in normal kidney but was upregulated in tubular cells of the kidneys after renal ischemia. Blockade of activin action by follistatin promoted tubular recovery after injury, thus suggesting that activin A is an endogenous inhibitor of tubular regeneration after injury.

Similarly to TGF-*β*, activin signaling is mediated by Smad2 and Smad3 [6]. Mice lacking Smad3 are protected against tubulointerstitial fibrosis following unilateral ureteral obstruction (UUO) by blocking of EMT and abrogation of monocyte influx and collagen accumulation [12], which suggests the involvement of activin signaling pathway in renal fibrosis. The present study demonstrated that the expression of activin A was significantly upregulated in the UUO kidneys and that recombinant follistatin prevented renal fibrosis in vivo. Blockade of activin action may therefore be a new strategy for the prevention of renal fibrosis progression.

## 2. Materials and Methods

### 2.1. Reagents

Recombinant human follistatin was provided by Dr. Y. Eto (Central Research Laboratory, Ajinomoto, Kawasaki, Japan). Antibodies used in this study were as follows: goat anti-type I collagen antibody (1 : 100), goat anti-type III collagen antibody (1 : 100) (Southern BioTech, Birmingham, AL), mouse anti-*α*-SMA antibody (1 : 100) (Sigma, St. Louis, MO), mouse anti-CD68 antibody (1 : 100) (Abcam, Cambridge, UK), rabbit anti-inhibin *β*A antibody (1 : 100) (Thermo Fisher Scientific, Yokohama, Japan), rabbit anti-fibronectin antibody (1 : 100), goat anti-vimentin antibody (1 : 100) (Santa Cruz biotechnology, Inc., CA), rabbit anti-CD3 antibody (1 : 100) (Vector Labs, Burlingame, CA), Alexa Fluor 488 goat anti-mouse IgG (1 : 2000), and Alexa Fluor 488 goat anti-rabbit IgG (1 : 2000) (Invitrogen, Carlsbad, CA).

### 2.2. Experimental Protocol

Male Wistar rats (200 g) were purchased from Nihon SLC Inc. (Hamamatsu, Japan). UUO was performed as described previously [13]. Briefly, after induction of general anesthesia by intraperitoneal injection of pentobarbital (50 mg/kg body wt), the abdominal cavity was exposed via a midline incision and the left ureter was ligated at three points with 4-0 silk. Recombinant human follistatin (1 *μ*g) or saline was administered intraperitoneally into rats at 1, 3, 5, and 7 days after UUO. At the indicated times after UUO, rats were sacrificed and the kidneys were removed for RNA extraction or histologic examination. UUO was confirmed by observation of dilation of the pelvis and proximal ureter and collapse of the distal ureter. Sham-operated kidneys without ligation were used as controls. The experimental protocol was approved by the Ethics Review Committee for Animal Experimentation of Gunma University.

### 2.3. Histological Examination

Kidneys were fixed in 10% formaldehyde and were embedded in paraffin. Sections (4 µm) were stained with periodic acid-Schiff (PAS) and Masson-trichrome (MT). MT-stained sections were microscopically examined and the changes observed were limited to the outer medulla, where fibrotic change is most obvious. For semiquantitative analysis, renal interstitial fibrosis was graded as follows: 0, 0%; 1, 0% to 25%; 2, 25% to 50%; 3, 50% to 75%; 4, 75% to 100% of involvement of microscopic field at ×400 magnification. Five sections from five rats (a total of 25 sections) were used for each condition. Data are expressed as mean ± SE (*n* = 5).

### 2.4. Cell Proliferation

Cell proliferation was assessed by in vivo DNA labeling with bromodeoxyuridine (BrdU), an analogue of thymidine. BrdU (100 mg/kg), which is incorporated into DNA during S phase of the cell cycle, was injected intraperitoneally into rats at 1 h before sacrifice. Kidneys were removed, fixed with formaldehyde, and embedded into paraffin. Sections were deparaffinized with xylene, rehydrated with graded ethanol solutions (100, 100, 90, 70, and 50%) for 10 min each, and washed twice with distilled water. BrdU-positive cells were detected using a Cell Proliferation Kit (Amersham Biosciences Corp., Piscataway, NJ), in accordance with the manufacturer's instructions.

Quantitative analysis of BrdU-positive cells was performed by counting the number of BrdU-positive cells in tubules and interstitium separately in 10 randomly selected fields at ×400 magnification.

### 2.5. Reverse-Transcription PCR (RT-PCR)

Whole kidneys were suspended in TRI reagent (Molecular Research Center Inc., Cincinnati, OH) and homogenized. Total RNA was extracted, and first-strand cDNA was prepared by reverse transcription with the Omniscript RT Kit (Qiagen Inc., Valencia, CA) using Oligo (dT) primer (Invitrogen Corp., Carlsbad, CA) according to the manufacturer's instructions. Reverse-transcription PCR (RT-PCR) was performed as described previously [13]. Sequences of primers used in this study were as follows: *β*A subunit (sense, 5′-GGACCTAACTCTCAGCCAGAGATG-3′; antisense, 5′-TCTCAAAATGCAGTGTCTTCCTGG-3′), activin receptor type I (sense, 5′-AGTCGTGGTTCAGGGAGACA-3′; antisense, 5′-GAGTGGTGAGCTGAAGGTAG-3′), activin receptor type II (sense, 5′-TGTGAAATGAGTAGGGTGCC-3′; antisense, 5′-CCTTCATATCCGTGTTGCAG-3′), follistatin (sense, 5′-AAAACCTACCGCAACGAATG-3′; antisense, 5′-AGGCATTATTGGTCTGATCC-3′), and GAPDH (sense, 5′-CTACCCACGGCAAGTTCAAT-3′; antisense, 5′-TACTCAGCACCAGCATCACC-3′). Quantitative real-time PCR was performed as described previously [14].

### 2.6. Immunohistochemical Analysis

Immunostaining with the avidin-biotin coupling immunoperoxidase technique was performed using a Vectastain Elite ABC kit (Vector Laboratories, Burlingame, CA) in accordance with the manufacturer's instructions. Briefly, sections were deparaffinized and rehydrated using standard methods. After inactivation of endogenous peroxidase with 1% metaperiodic acid in phosphate-buffered saline (PBS) for 10 min at room temperature, sections were preincubated with 3% BSA-PBS for 1 h. Sections were then incubated with primary antibody for 2 h, washed with PBS, and reacted with a biotinylated secondary antibody for 1 h. After washing with PBS, sections were reacted with Vectastain Elite ABC reagent. Antibody was detected with diaminobenzidine tetrahydrochloride in PBS, and sections were counterstained with hematoxylin. For immunohistochemical controls, primary antibody was replaced with 3% BSA-PBS, which did not exhibit positive staining, thus confirming specificity.

Indirect immunofluorescence staining was performed as described previously [14]. Briefly, frozen sections were washed in PBS, pretreated with 3% BSA-PBS for 1 h, and covered with primary antibody at room temperature for 1 h. After washing in PBS, sections were covered with a mixture of a fluorescence-labeled secondary antibodies and 4′-diamidino-2-phenylindole (DAPI). Immunofluorescence images were recorded with a Spot RT Slider digital camera attached to a Nikon Eclipse 80i fluorescence microscope.

Quantification of the positive area for *α*-SMA, type I collagen, type III collagen, and fibronectin was calculated by image analysis using Image J software (NIH, Bethesda, MD, USA). The mean value of the positive area was obtained by evaluating 10 randomly selected fields at ×200 magnification per kidney.

### 2.7. Statistical Analysis

Differences in means between groups were compared by Student's* t*-test, and *P* values of <0.05 were considered to be significant.

## 3. Results

### 3.1. Expression of Activin A, Activin Receptors, and Follistatin in Kidneys after UUO

**Figure 1:**
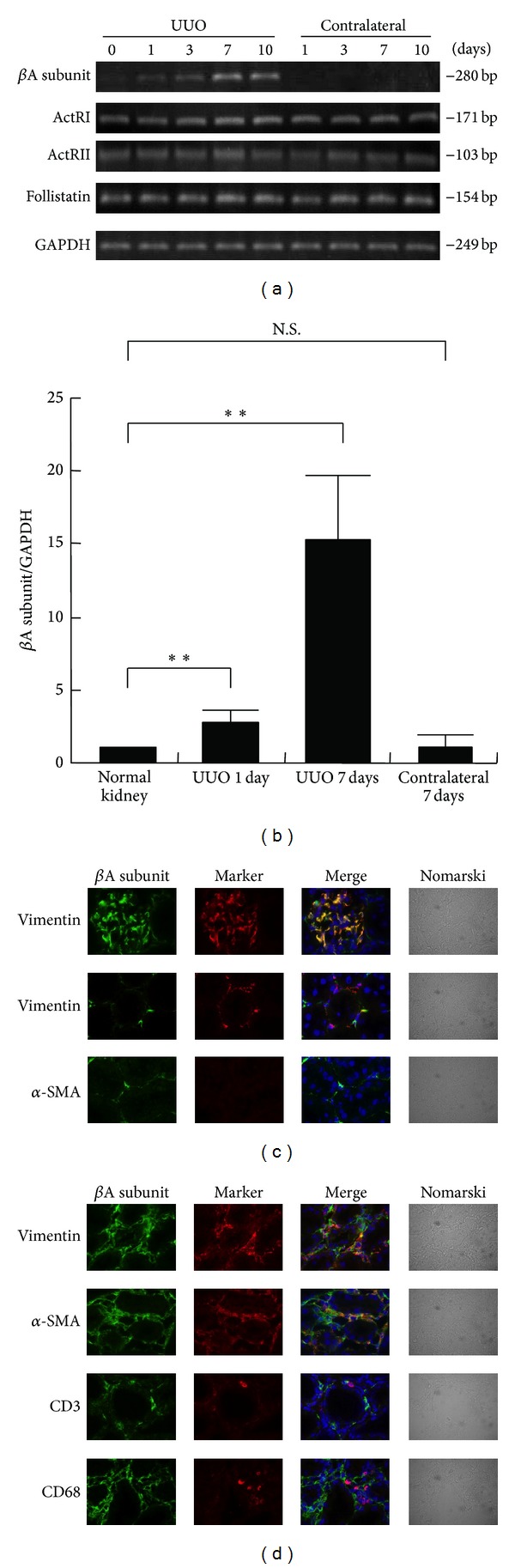
Expression of activin A, activin receptors, and follistatin in kidneys after UUO. (a) Total RNA was extracted from contralateral and UUO kidneys at the indicated times after surgery. Expression of *β*A subunit for activin A, activin type I receptor (ActRI), activin type II receptor (ActRII), and follistatin was examined by RT-PCR. (b) Expression of *β*A subunit for activin A in kidneys after UUO was measured by real-time PCR. Data are presented as mean ± SE (*n* = 3). **P < 0.01 versus normal kidney. N.S., not significant. (c) Localization of *β*A subunit for activin A in normal kidneys was examined by immunostaining. Magnification: ×1000. (d) Localization of *β*A subunit for activin A in the kidneys at 7 days after UUO was examined by immunostaining. Magnification: ×1000.

We first examined the expression of the *β*A subunit for activin A, activin receptors, and follistatin in UUO kidneys by RT-PCR. Expression of *β*A subunit mRNA was undetectable in normal, sham-operated (data not shown), and contralateral kidneys (Figure 1(a)). In contrast, *β*A subunit expression was observed in the UUO kidneys at 1 day and thereafter. Activin signals are known to be mediated through two types of activin receptors, type I (ActRI) and type II (ActRII) [6]. Consistent with previous data showing that both of these activin receptors are localized in the tubular cells of kidneys [15], expression of both ActRI and ActRII is detectable in normal, sham-operated, contralateral, and UUO kidneys. Expression of follistatin, an activin antagonist, was also present in normal, sham-operated, contralateral, and UUO kidneys. Quantitative real-time PCR confirmed that the expression levels of *β*A subunit mRNA were significantly elevated in UUO kidneys, as compared to those in normal or contralateral kidneys (Figure 1(b)). There were no significant differences in the expression levels of activin receptors or follistatin among normal, sham-operated, contralateral, and UUO kidneys (data not shown).

We also investigated the localization of activin A in the UUO kidney by immunostaining. Immunoreactive activin A was detected in vimentin-positive glomerular mesangial cells in normal (Figure 1(c), upper panels), sham-operated, and contralateral kidneys (data not shown). Vimentin-positive interstitial fibroblasts also produce activin A in normal kidneys (Figure 1(c), middle panels). The expression of *α*-SMA was undetectable in normal kidneys (Figure 1(c), bottom panels) except in vascular smooth muscle cells (data not shown). In contrast, numerous *α*-SMA-positive cells were observed in the interstitium of UUO kidneys (Figure 1(d)). In UUO kidneys, activin A was colocalized with interstitial vimentin-positive fibroblasts or *α*-SMA-positive myofibroblasts, but not with CD3-positive T lymphocytes or CD68-positive macrophages (Figure 1(d)). These results suggest that interstitial fibroblasts are the activin-producing cells in the kidney after UUO.

### 3.2. Effects of Follistatin on Fibrotic Change of the Kidneys after UUO

**Figure 2:**
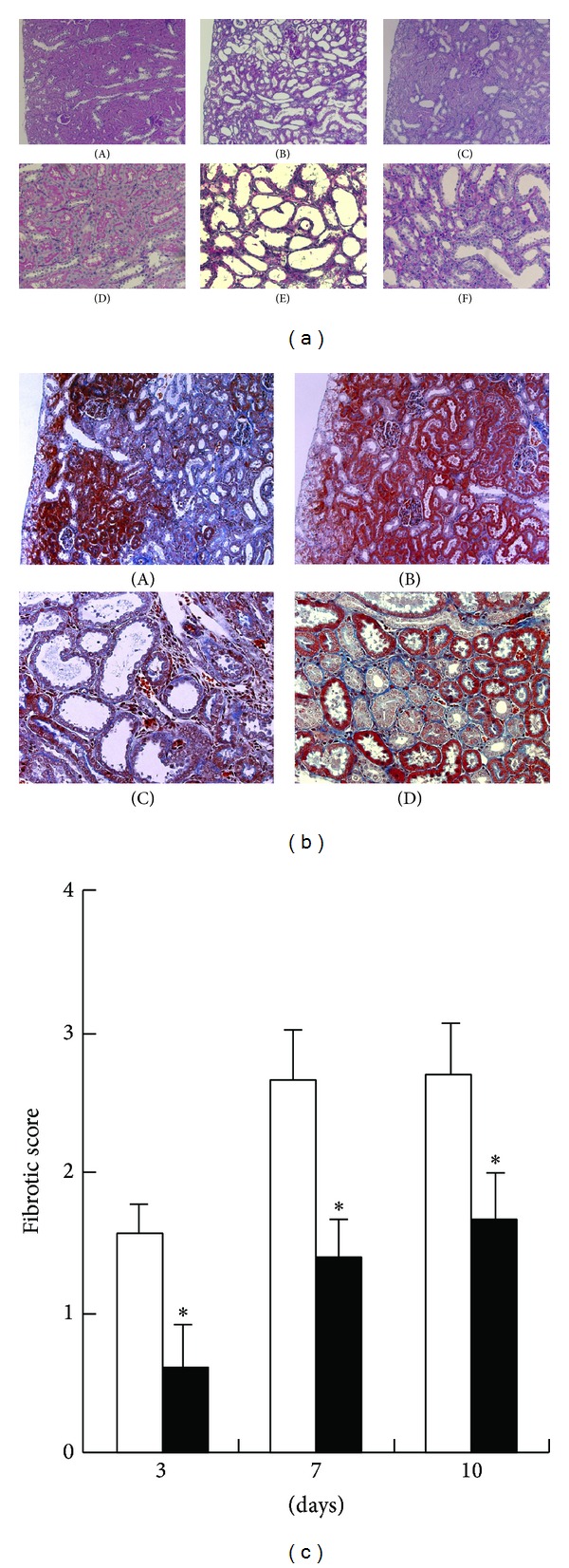
Effects of follistatin on fibrotic changes in kidneys after UUO. (a) Histological changes in kidneys after UUO were assessed by PAS staining. (A, D) contralateral kidneys, 7 days. (B, E) saline-treated UUO kidneys, 7 days. (C, F) follistatin-treated UUO kidneys, 7 days. Magnification: ×100 (A–C), ×400. (b) Fibrotic changes in kidneys after UUO were assessed by Masson-trichrome staining. (A, C) saline-treated UUO kidneys, 7 days. (B, D) follistatin-treated UUO kidneys, 7 days. Magnification: ×100 (A, B), ×400 (C, D). (c) Semiquantitative analysis of fibrotic changes in UUO kidneys. Fibrotic score was measured as described in Section 2. Data are presented as mean ± SE (*n* = 5). Saline (white bars), follistatin (black bars). *P < 0.05 versus saline.

In order to examine the role of endogenous activin A in this model, we administered recombinant human follistatin into the UUO-operated rats and assessed the effects of activin blockade on histological changes of the kidneys after UUO (Figure 2). PAS staining demonstrated normal architecture in normal, sham-operated (data not shown), and contralateral kidneys (Figure 2(a), panels (A) and (D)). Tubular dilation and atrophy were observed in the saline-treated UUO kidneys (Figure 2(a), panels (B) and (E)). In the follistatin-treated UUO kidneys, the renal parenchyma was markedly preserved (Figure 2(a), panels (C) and (F)). Masson-trichrome staining revealed the presence of interstitial fibrotic changes in the saline-treated UUO kidneys (Figure 2(b), panels (A) and (C)), but not in normal or contralateral kidneys (data not shown). The interstitial fibrotic area in the follistatin-treated kidneys was slightly reduced when compared to that in the saline-treated kidneys (Figure 2(b), panels (B) and (D)). Semiquantitative analysis showed that the fibrotic score of follistatin-treated kidneys was significantly lower than that of saline-treated kidneys (Figure 2(c)).

### 3.3. Effects of Follistatin on Cell Proliferation in UUO Kidneys

**Figure 3:**
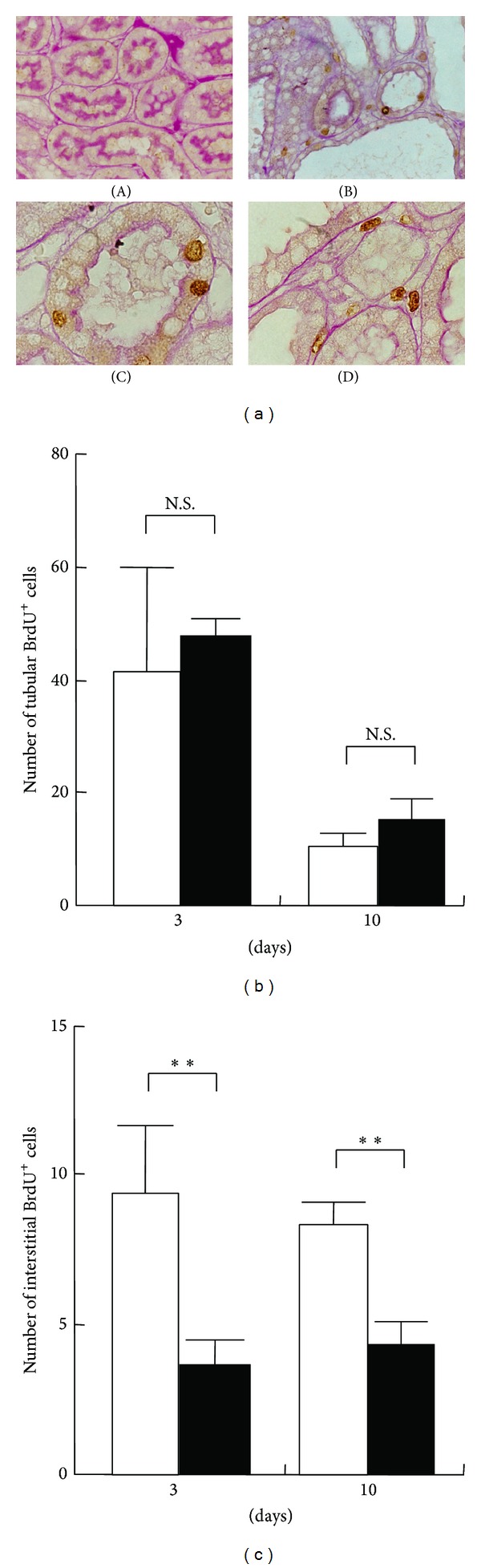
Effects of follistatin on cell proliferation in kidneys after UUO. (a) BrdU was intraperitoneally injected into UUO rats at 1 h before sacrifice. Cell proliferation was assessed by BrdU incorporation. (A) Contralateral kidneys, 3 days. UUO kidneys, 3 days. Magnification: ×200 (A, B), ×1000 (C, D). BrdU-positive nuclei (brown). (b), (c) Quantitative analysis of the number of tubular (b) and interstitial (c) BrdU-positive cells. BrdU-positive cells in the tubules and interstitium of the kidneys were separately counted in 10 randomly selected fields per rat at ×400 magnification. Values are mean ± SE (*n* = 5). Saline (open circle), follistatin (closed circle). **P < 0.01 versus saline. N.S., not significant.

Cell proliferation was assessed by BrdU incorporation (Figure 3). BrdU-positive cells were rarely observed in normal (data not shown) or contralateral kidneys (Figure 3(a), panel (A)). In contrast, a large number of BrdU-positive cells were observed in UUO kidneys on day 3 (Figure 3(a), panel (B)). Most BrdU-positive cells were localized in tubular cells (Figure 3(a), panel (C)) and some were present in the interstitium of UUO kidneys (Figure 3(a), panel (D)). Quantitative analysis showed that there was no significant difference in the number of BrdU-positive tubular cells between saline-treated and follistatin-treated kidneys (Figure 3(b)). Interestingly, the number of BrdU-positive interstitial cells was significantly lower in the follistatin-treated kidneys, as compared to saline-treated kidneys (Figure 3(c)).

### 3.4. Effects of Follistatin on the Expression of *α*-SMA in UUO Kidneys

**Figure 4:**
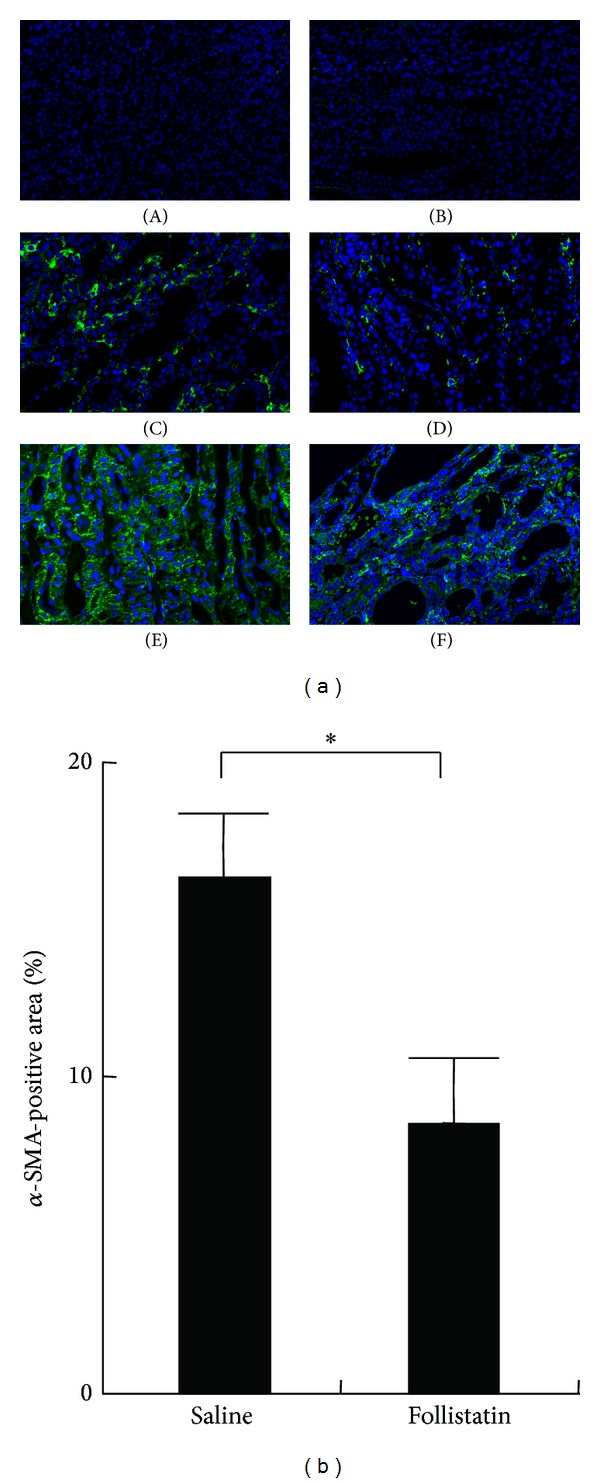
Effects of follistatin on expression of *α*-SMA in kidneys after UUO. (a) Expression of *α*-SMA, a marker for myofibroblasts, in the UUO kidneys, was examined by immunostaining. (A) Normal kidney. (B) Contralateral kidneys, 10 days. (C) Saline-treated UUO kidneys, 3 days. (D) Follistatin-treated UUO kidneys, 3 days. (E) Saline-treated UUO kidneys, 10 days. (F) Follistatin-treated UUO kidneys, 10 days. Magnification: ×200. *α*-SMA (green), DAPI (blue). (b) Quantitative analysis of *α*-SMA-positive area. *α*-SMA-positive area in the kidneys at 10 days after UUO was assessed as described in Section 2. Values are mean ± SE (*n* = 5). *P < 0.05.

Transdifferentiation of interstitial fibroblasts into myofibroblasts is one of the critical processes involved in renal fibrosis [3]. We next investigated the expression of *α*-SMA, a marker of myofibroblasts, in UUO kidneys. No expression of *α*-SMA was observed in normal (Figure 4(a), panel (A)) and contralateral kidneys (Figure 4(a), panel (B)) except in vascular smooth muscle cells. In contrast, expression of *α*-SMA was abundantly detected in the interstitium of the saline-treated UUO kidneys (Figure 4(a), panels (C) and (E)). In the follistatin-treated kidneys, *α*-SMA expression was also observed (Figure 4(a), panels (D) and (F)), but its positive area was significantly smaller than that in the saline-treated kidneys at day 7 after UUO (Figure 4(b)).

### 3.5. Effects of Follistatin on Extracellular Matrix Production in UUO Kidneys 

**Figure 5:**
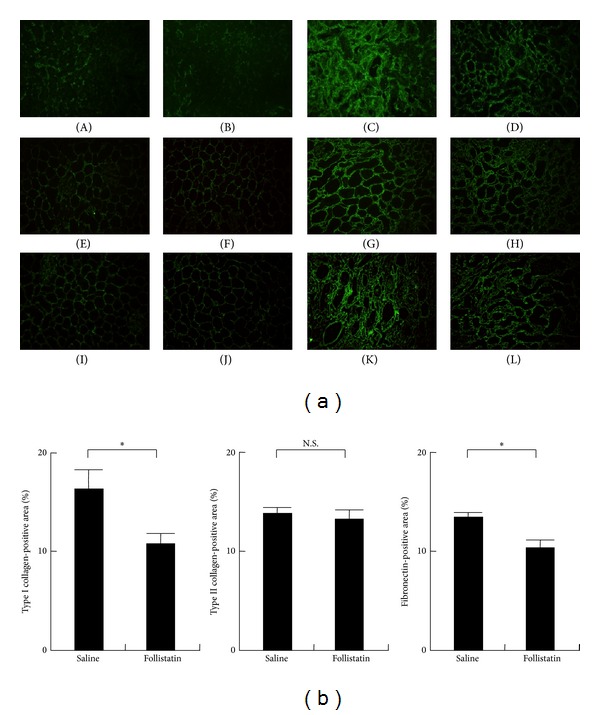
Effects of follistatin on the production of extracellular matrix in kidneys after UUO. (a) Production of type I collagen (A–D), type III collagen (E–H), and fibronectin (I–L) in the UUO kidneys was examined by immunostaining. (A, E, I) normal kidney. (B, F, J) contralateral kidney. (C, G, K) saline-treated UUO kidney, 7 days. (D, H, L) follistatin-treated UUO kidney, 7 days. Type I collagen, type III collagen, and fibronectin (green). Magnification: ×100. (b) Quantitative analysis of extracellular matrix production. Type I collagen, type III collagen, and fibronectin-positive area in kidneys at 7 days after UUO was measured as described in Section 2. Values are mean ± SE (*n* = 5). *P < 0.05. N.S., not significant.

Myofibroblasts produce various types of extracellular matrix (ECM), leading to the deposition of ECM during renal fibrosis. We next examined the effects of follistatin on the production of ECM by immunostaining (Figure 5). The deposition of type I collagen (Figure 5, panels (A) to (D)), type III collagen (Figure 5, panels (E) to (H)), and fibronectin (Figure 5, panels (I) to (L)) was observed in both saline-treated (Figure 5, panels (C), (G), and (K)) and follistatin-treated kidneys (Figure 5, panels (D), (H), and (L)), but not in normal (Figure 5, panels (A), (E), and (I)) or contralateral kidneys (Figure 5, panels (B), (F), and (J)). Quantitative analysis showed a significant decrease in type I collagen-positive area as well as fibronectin-positive area, but not in type III-positive area in the follistatin-treated kidneys (Figure 5(b)).

### 3.6. Effects of Follistatin on Macrophage Infiltration in UUO Kidneys

**Figure 6:**
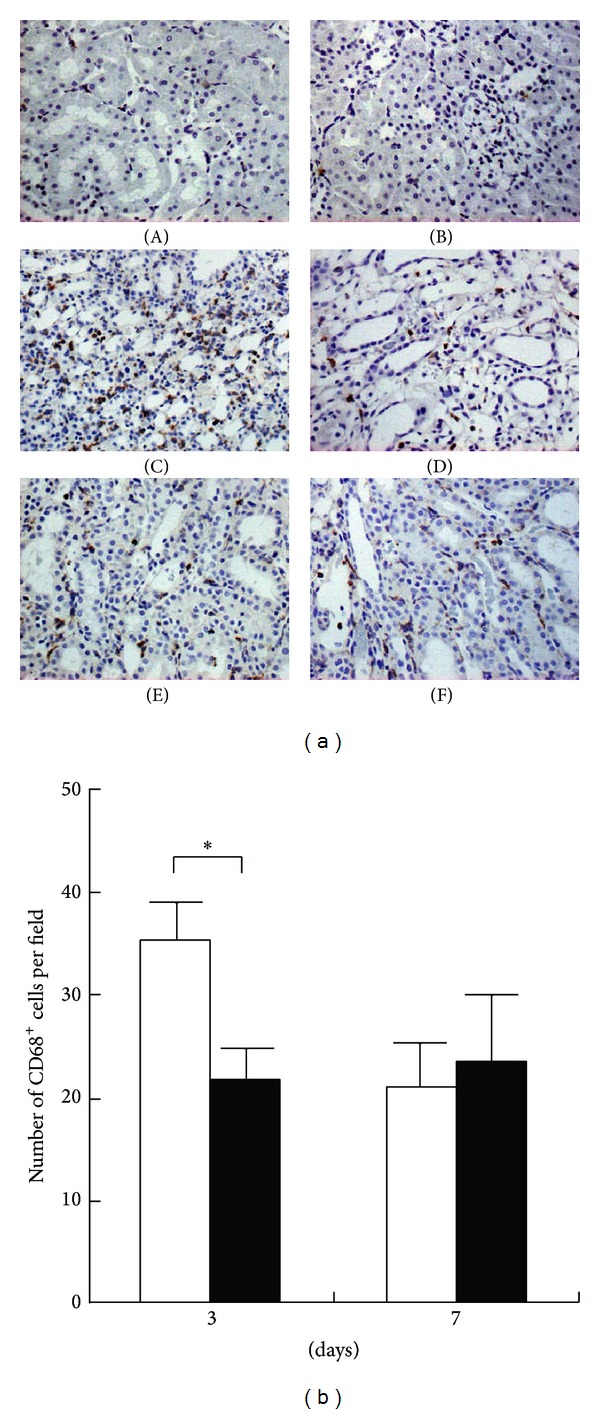
Effects of follistatin on macrophage infiltration in kidneys after UUO. (a) Expression of CD68, a marker for macrophages, in UUO kidneys, was examined by immunostaining. (A) Normal kidney. (B) Contralateral kidneys, 3 days. (C) Saline-treated UUO kidneys, 3 days. (D) Follistatin-treated UUO kidneys, 3 days. (E) Saline-treated UUO kidneys, 7 days. (F) Follistatin-treated UUO kidneys, 7 days. CD68-positive cells (brown). Magnification: ×400. (b) Quantitative analysis of CD68-positive cell number. CD68-positive cells were counted in 10 randomly selected fields per rat at ×400 magnification. Values are mean ± SE (*n* = 5). Saline (white bars), follistatin (black bars). *P < 0.05 versus saline.

Macrophage infiltration is often correlated with degree of renal fibrosis, and the depletion of macrophages reduces fibrosis in several disease models [16]. We then investigated macrophage infiltration in UUO kidneys by immunostaining. The expression of CD68, a macrophage marker, was not observed in normal (Figure 6(a), panel (A)) or contralateral kidneys (Figure 6(a), panel (B)). In contrast, CD68-positive cells were observed in the interstitium of saline-treated UUO kidneys (Figure 6(a), panels (C) and (E)) and follistatin-treated UUO kidneys (Figure 6(a), panels (D) and (F)). Semiquantitative analysis demonstrated that the number of CD68-positive cells in follistatin-treated kidneys was significantly lower when compared to that in the saline-treated kidneys at day 3, but not at day 7, after UUO (Figure 6(b)).

## 4. Discussion

Activin A is involved in tissue fibrosis in various organs [17]. Expression of activin A is upregulated in the fibrotic process in several tissues, including the lung [18, 19], pancreas [20], liver [21], and skin [22, 23]. Follistatin attenuated early events in fibrogenesis by constraining hepatic satellite cell proliferation and inhibiting hepatocyte apoptosis [24]. Furthermore, follistatin exerted antifibrotic effects in bleomycin-induced pulmonary fibrosis [25]. In transgenic mice overexpressing follistatin in the epidermis, scar formation was decreased after wounding the skin [26]. However, the role of activin A in renal fibrosis is unclear. We demonstrated here the upregulation of activin A in the UUO kidneys. Immunoreactive activin A was abundantly expressed by *α*-SMA-positive interstitial myofibroblasts in the UUO kidneys (Figure 1). Blockade of activin by follistatin reduced the fibrotic changes (Figure 2) and reduced the production of type I collagen and fibronectin (Figure 5) in the kidneys after UUO. Furthermore, follistatin inhibited the number of interstitial proliferating cells (Figure 3) and significantly reduced *α*-SMA-positive area in the UUO kidneys (Figure 4). It was previously demonstrated that activin A promoted cell proliferation and increased the production of both type I collagen and *α*-SMA expression in primary renal fibroblasts in vitro [13]. Renal interstitial fibroblasts express activin receptors [13]. These data suggest that activin A serves as an autocrine inducer of cell proliferation or activator of interstitial fibroblasts. The activin signaling pathway may be a novel therapeutic target for the prevention of renal fibrosis.

The precise mechanism by which follistatin reduced renal fibrosis remains unclear in this study. Follistatin may antagonize activin A action by blocking interaction with activin receptors on fibroblasts and preventing downstream signaling cascades leading to extracellular matrix synthesis. In addition to antagonizing the profibrotic action of activin A, two mechanisms may explain the therapeutic effects of follistatin on renal fibrosis. First, follistatin attenuates renal fibrosis by blocking the action of TGF-*β*. It was reported previously that activin A expression is induced by TGF-*β*1 and that blockade of activin by follistatin or transfection with truncated type II activin receptor reduces type I collagen expression induced by TGF-*β*1 in rat primary renal fibroblasts [13]. Similarly, TGF-*β*1 activity was inhibited by blockade of activin in rat hepatic stellate cells [24], pancreatic stellate cells [20], and human fetal lung fibroblasts [25]. It is therefore likely that TGF-*β*1 induces tissue fibrosis partly via activin A. Second, the action of follistatin is mediated through other members of the TGF-*β* superfamily. Follistatin binds to activin with high affinity and also binds to several BMP proteins [27]. Follistatin enhanced BMP-7 action to induce muscle growth during chick limb development [28]. Systemic administration of recombinant human BMP-7 leads to the repair of severely damaged renal tubular epithelial cells, in association with reversal of chronic renal injury [29]. Therefore, amelioration of renal fibrosis by follistatin may be obtained by the enhancement of the antifibrotic effects of BMP-7.

Emerging evidence has suggested activin A as a key mediator in inflammation [30]. Activin A exhibits proinflammatory actions in several tissues [17], is secreted from activated immune cells recruited to sites of inflammation by mast cell progenitors [31], and induces the directional migration of immature myeloid dendritic cells through the activation of activin receptors [32]. In the colitis model, infiltrating macrophages were found to produce excess activin *β*A [33]. Immunoreactive activin A was abundantly expressed in the infiltrated macrophages in bleomycin-treated rat lung [25]. CD68-positive macrophage-lineage cells have been identified as activin-producing cells in rheumatoid synovium [34]. In an inflammatory state, activin A may be involved in the infiltration of macrophages by stimulating the gelatinolytic activity of matrix metalloproteinase-2 (MMP-2), which can degrade basement membrane collagens [35]. In this study, as an early inflammatory response after UUO, the infiltration of CD68-positive macrophages was observed in the UUO kidneys. Although it is unknown whether this inflammatory response directly contributes to the fibrotic process in this UUO model, follistatin significantly reduced the number of infiltrating CD68-positive macrophages (Figure 6). Activin A was expressed in the interstitial fibroblasts but not colocalized with infiltrating macrophages (Figure 1(d)). This raises the possibility that activin A, as a chemoattractant, may help macrophages infiltrate the interstitium during renal fibrosis. Decrease in the number of CD68-positive cells by follistatin could be observed at 3 days but not at 7 days after UUO (Figure 6), which might suggest the presence of chemoattractants other than activin A or activin-independent mechanism. Further study is necessary to clarify this issue.
